# Affect, risk perception, and the use of cigarettes and e-cigarettes: a population study of U.S. adults

**DOI:** 10.1186/s12889-018-5306-z

**Published:** 2018-03-22

**Authors:** Lucy Popova, Daniel Owusu, Scott R. Weaver, Catherine B. Kemp, C. K. Mertz, Terry F. Pechacek, Paul Slovic

**Affiliations:** 1 School of Public Health and Tobacco Center of Regulatory Science, Georgia State University, P.O. Box 3995, Atlanta, GA 30302-3995 USA; 2 Decision Research, 1201 Oak Street, Suite 200, Eugene, OR 97401 USA; 3 Department of Psychology, University of Oregon, Eugene, OR USA

**Keywords:** Affect, Risk perception, Tobacco, Electronic cigarettes, Cigarettes, Smoking

## Abstract

**Background:**

Tobacco companies argue that the decision to smoke is made by well-informed rational adults who have considered all the risks and benefits of smoking. Yet in promoting their products, the tobacco industry frequently relies on affect, portraying their products as part of a desirable lifestyle. Research examining the roles of affect and perceived risks in smoking has been scant and non-existent for novel tobacco products, such as electronic cigarettes (e-cigarettes).

**Methods:**

We examined the relationship between affect, perceived risk, and current use for cigarettes and e-cigarettes in 2015 in a nationally representative sample of 5398 U.S. adults who were aware of e-cigarettes.

**Results:**

Participants held various affective associations with tobacco products, and affect towards cigarettes was more negative than affect towards e-cigarettes. Using structural equation modeling (SEM), affect towards cigarettes and e-cigarettes was associated with cigarette smoking and e-cigarette use respectively, and these associations were both direct and partially mediated by risk perceptions towards smoking and e-cigarette use. More positive affect towards cigarettes or e-cigarettes was associated with lower perceived risks, which in turn was associated with higher odds of being a current cigarette or e-cigarette user.

**Conclusions:**

In developing models explaining tobacco use behavior, or in creating public communication campaigns aimed at curbing tobacco use, it is useful to focus not only on the reason based predictors, such as perceptions of risks and benefits, but also on affective predictors. Educational efforts aimed at further smoking reductions should highlight and reinforce negative images and associations with cigarettes.

**Electronic supplementary material:**

The online version of this article (10.1186/s12889-018-5306-z) contains supplementary material, which is available to authorized users.

## Background

Despite over 50 years of awareness that smoking causes cancer and premature death, tobacco use remains the leading cause of preventable disease and death in the United States [1, 2]. While the prevalence of smoking has declined in recent years in the United States, over 36 million adults still are current smokers [3]. Why do people start or quit smoking? The answers to this question come from two domains: cognition and affect. Answers from the cognitive domain focus on conscious and deliberate thought processes, such as stated perceptions of risk and benefits [4–6]. In contrast, answers from the affective theories highlight the importance of feelings associated not only to reasons but to all cognitive content, including thoughts and images that influence us in ways that we are not consciously aware of [7]. Affect here is defined as a positive or a negative feeling about an object or a stimulus. Affective reactions can be almost instantaneous and can occur with or without conscious awareness [7].

The decision to start, continue, or stop smoking has sometimes been conceptualized as a decision arrived at by a perfectly or imperfectly rational [8] person who weighs the costs and benefits of the decision and calculates the optimal behavior [9]. This is the view tobacco companies have long been trying to promulgate. Defending themselves in court, tobacco companies have denied that nicotine is addictive [10] and have argued that they should not be blamed for the deaths and diseases caused by smoking because the decision to smoke was made by a rational, well-informed adult who knew and willingly accepted the risks of smoking [1, 11, 12]. Yet in their advertising, tobacco companies have employed quite a different strategy. They focus on affect instead of the analytic perception of risk.

Tobacco companies have long understood the importance of affect in promoting smoking [12]. As the growing scientific knowledge in the 1950s and 1960s led to increased awareness of the serious health effects of smoking, tobacco companies started promoting products aimed at reducing perceptions of risk [13], such as filtered and low-tar cigarettes [14]. The early advertisements for low-tar cigarettes focused on reasons and arguments based on features such as machine yields and amount of tar [15]. These early advertisements were designed to appeal to the analytical processing system in smokers [16, 17]. However, tobacco companies were concerned that this approach was insufficient to allay the worries of health-conscious consumers who might quit completely rather than use low-tar cigarettes [14]. These reason-based advertisements reminded smokers about health risks and evoked more negative feelings than they alleviated [14]. Thus, on the advice of their marketing consultants, the companies switched to appealing to positive feelings instead, generating favorable imagery of smoking and smokers through use of pictures and associations with highly desirable places and situations, such as sportiness, sophistication, style, and relaxation [15].

What tobacco companies have realized is that current and potential cigarette smokers are driven by affect to a greater extent than they are driven by calculations of risk and benefit [18, 19]. More generally, behavioral research has increasingly recognized the role of affect in decision making, particularly around risky behavior. There are several conceptualizations of the role of affect in judging the risk and acting on it, such as risk-as-feeling hypothesis [20], affect heuristic [21], and somatic markers [22]. All have in common the notion that representations of objects and events in our minds are inextricably linked with positive or negative feelings (which are connected to body or somatic states, as Damasio argued). In making a judgment, people refer to these associated feelings, and they serve as shortcuts for quick decision making.

Research comparing the differential effects of affective and rational perceptions has found that affect is a better predictor of smoking compared to reasoned perceptions or instrumental beliefs [23, 24]. These studies considered affect and cognitive beliefs as independent predictors of behavior without examining the (potentially causal) association between them. However, according to the affect heuristic [21] and the risk as feeling hypothesis [20], the affective, largely automatic output of the experiential system precedes and guides analytical reasoning [25, 26]. Several studies tested a mediated path model and found that affect predicted smoking intentions and behavior directly and indirectly through perceptions of risks and benefits [27, 28]. All of the above studies were limited to convenience samples and looked exclusively at cigarette smoking.

With smoking rates declining, tobacco companies have been looking for ways to reinvigorate their revenues. Electronic cigarettes (also called “vapes”, “e-cigs”, ENDS, e-cigarettes) are, according to some researchers, a “disruptive technology” [29–31] that holds the answer to eradicating cigarettes or at least reducing harms of smoking. Other scholars have argued that e-cigarettes have yet to meet the disruptive technology threshold since a majority of smokers find them to be a less satisfying alternative [32]. Some scholars have expressed concern that e-cigarettes have the potential to renormalize smoking [33] and might make it harder for smokers to quit [34]. Evidence from tobacco industry documents show that tobacco industry has researched nicotine aerosol technology similar to modern e-cigarettes since 1990s as a complementary rather than a competing “disruptive” technology to cigarettes in an effort to deter health conscious smokers from using nicotine replacement therapy to quit smoking [35]. Today, every major tobacco company offers an electronic cigarette product (MarkTen – Altria; Vuse – RJ Reynolds; blu – first purchased by Lorillard, now owned by Imperial Brands) and the industry is consolidating around these new products and marketing models [36]. E-cigarettes’ advertising has spun the gamut of cognitive and emotional appeals, from portraying e-cigarettes as safer and healthier than cigarettes, being a “resolution solution” (NJOY) and a choice recommended by doctors to creating positive images of rebellious e-cigarette users who “take back” their freedom (blu) and “rewrite the rules” (Fin) [37, 38].

Recent research has examined how perceptions of risk and other cognitive factors are associated with initiation, use, and discontinuation of electronic cigarettes [39–41]. While some studies have looked at the role affect might play in sparking interest in electronic cigarettes [42], none examined how affect and risk perceptions of e-cigarettes are associated in relation to e-cigarette use.

In this paper, we used a nationally representative sample to examine how current, former, and never users of cigarettes and e-cigarettes feel about cigarettes and e-cigarettes and what affective imagery they associate with each product. We hypothesized that more positive affect is related to being a current user of the product. We also evaluated whether the pattern of associations among affect, risk perceptions, and product use for cigarettes and e-cigarettes is consistent with a mediational model derived from the theoretical framework of the affect heuristic and “risk as feelings” whereby affect has an indirect effect on product use through risk perceptions.

## Methods

### Data source

We analyzed data from the Tobacco Products and Risk Perceptions Survey (TPRPS) conducted from August to September 2015 by the Georgia State University Tobacco Center of Regulatory Science (TCORS). An annual cross-sectional survey, TPRPS is administered to a probability sample drawn from GfK’s KnowledgePanel, a probability-based web panel designed to be representative of non-institutionalized USA adults. KnowledgePanel only includes adults sampled via address-based sampling. Participants lacking Internet access are provided a computer to facilitate participation. A sample of 6091 qualified completers was obtained from 8135 KnowledgePanel members who were invited to participate in the 2015 survey. The final sample of 6051 cases with a final stage completion rate of 76.0% and a study qualification rate of 98.5% was achieved, after exclusion of 40 cases due to non-response to more than one-half of the survey questions. The analytic sample for this study comprised 5389 participants who reported being aware of any kind of electronic vaporizer product (“Have you ever seen or heard of any type of electronic vapor product, such as e-cigarettes, e-cigars, e-hookahs, e-pipes, vape pens, hookah pens or personal vaporizers/mods before this study?”). Participants who reported not being aware were excluded because they were not asked questions about e-cigarettes. We used an iterative proportional fitting (raking) procedure to adjust for sources of sampling and non-sampling error (such as panel recruitment non-response and panel attrition) to compute a study-specific post-stratification weight. Demographic and geographic distributions from the March 2015 Current Population Survey (CPS) served as benchmarks for adjustment, and included sex, age, race/ethnicity, education, household income, census region, metropolitan area, and internet access. TPRPS was approved by the Georgia State University Institutional Review Board.

### Measures

#### Affect towards cigarettes and e-cigarettes

Participants were asked, “When you hear the word cigarette, what is the first thought or image that comes to mind? Please list just one thought or image.” The same question was used for e-cigarettes, but the word “cigarette” was replaced by the phrase “electronic vapor products, which includes e-cigarettes, e-cigars, e-hookahs, e-pipes, vape pens, hookah pens and personal vaporizers/mods.” (Before any questions about e-cigarettes, the survey provided an explanation of what e-cigarettes are, accompanied by a picture illustrating various e-cigarette types and devices.) Participants were then asked, “How do you feel about this thought or image? Please rate this thought or image and not the word “cigarette” [“electronic vapor product”] itself.” They could rate it on a 5-point scale from − 2 (very bad) to + 2 (very good) (with 0 being “neither good nor bad”). The same sets of questions were repeated for the second thought or image for both products. In the present study the correlation across participants between the two affect ratings was *r* = .86 for cigarette images and *r* = .85 for e-cigarette images. We do not differentiate between thoughts or images and refer to them as “images” for the remainder of the paper. This measure of affect has been previously validated by studies showing that the qualitative nature of the images and their valence contribute to an understanding of the meaning of the target concept to an individual and predict a diverse range of judgments, decisions, and behaviors [43, 44].

#### Risk perceptions of cigarettes and e-cigarettes

To measure perceived risks, participants were asked “Imagine that you just began [smoking cigarettes / using electronic vapor products] every day. What do you think your chances are of having each of the following happen to you if you continue to [smoke cigarettes / use electronic vapor products] every day?” with respect to the following conditions: lung cancer, lung disease other than lung cancer (such as COPD and emphysema), heart disease, and premature death. Response options included a seven-point Likert-type scale ranging from 0 (“No chance”) to 6 (“Very good chance”), and a separate “I don’t know” category, which was treated as missing data in our analyses.

#### Outcome measures

Behavioral outcomes were: (1) smoking status and (2) e-cigarette use. Smoking status was categorized as never smokers (have not smoked 100 cigarettes in their lifetime), current smokers (smoked 100 cigarettes or more and currently smoking every day or some days), and former smokers (smoked 100 cigarettes or more but currently not smoking). E-cigarette use was categorized as never users (never tried e-cigarettes), current users (currently using e-cigarettes every day, some days, or rarely), and former users (have tried e-cigarettes but not currently using them at all).

#### Demographics

We measured sex, age, race, and education, categorizing them as shown in Table 5.

### Data analytic approach

We conducted the analyses for this study in two stages using Mplus statistical software (v. 7.4) [45]. In the initial stage, we used ordinal confirmatory factor analysis (CFA) [46] to examine the factorial validity of the measurement models for the a*ffect* and r*isk perceptions* constructs, separately for each construct and followed by a correlated factors confirmatory factor model that incorporated factors for each construct (and each product – cigarettes and e-cigarettes). A mean- and variance-adjusted weighted least squares estimator (WLSMV) was used for these CFA models. Model fit was assessed by examination of the chi-square test of exact fit; approximate fit indices (viz., root mean square error of approximation [RMSEA] and comparative fit index [CFI], using criteria suggested by Hu & Bentler) [47]; magnitude and consistency of factor loadings; and modification indices.

In the second stage, we used structural equation modeling (SEM) to estimate the parameters of the hypothesized mediation model of the effect of a*ffect* on tobacco product *use* (cigarettes / e-cigarettes) via *perceived risk* of the product, while adjusting for covariates (gender, age, race/ethnicity, and education). A logit link function was used to model the multinomial log-odds of product use (current use = referent category) as a function of *affect*, *risk perceptions*, and covariates. These analyses employed a robust, full information maximum likelihood estimator (MLR) and adaptive numerical integration (trapezoid; 50 integration points per dimension) with expectation-maximization algorithm.

All analyses reported were weighted to account for the complex sampling design and generate estimates generalizable to the subpopulation of U.S. adults who are aware of e-cigarettes. Participants with missing data are included in the analysis if they have at least one non-missing data point under the assumption of *missing at random* for the full-information MLR estimator and *missing at random given observed covariates* for the WLSMV estimator [48]. Responses of “don’t know” for the risk perception items were modeled as missing data for all analyses. The amount of missing data for this study was minimal. The median covariance coverage, the proportion of cases that provide complete data for a pair of variables, across all pairs of variables in the models was 91.1%. All statistical tests and confidence intervals were two-tailed with alpha = .05.

## Results

### Demographic characteristics

Of those aware of e-cigarettes, 50.9% were females, 21.2% were 18–29 years old, 25.3% were 30–44, 27.5% were 45–59, and 26.0% were 60 years and older; 68.1% were White, non-Hispanic, 10.4% were Black, non-Hispanic, 14.6% were Hispanic, and 6.9% other race. Additionally, 10.67% had below high school education, 29.1% had high school diploma, 29.2% had some college education, and 31.1% had bachelor’s degree or higher education. Current smokers comprised an estimated 14.8% of the study population, 28.4% were former smokers, and 57.4% were never smokers. Approximately 8.5% were current e-cigarette users, 11% were former users, and 80.6% had never tried e-cigarettes. As reported elsewhere, 29.8% of current smokers were dual users of e-cigarettes (among current e-cigarette users, 56.9% were current smokers) [49] (see Additional file 1: Table S1).

### Risk perceptions

Mean scores for cigarette risk perception ranged from 5.30 (heart disease) to 5.41 (lung disease other than lung cancer), and those of e-cigarette risk perception ranged from 4.17 (premature death) to 4.30 (lung disease other than lung cancer) (see Additional file 2: Table S2).

### Affective imagery

Participants provided 9900 images of cigarettes and 9747 images for e-cigarettes. We coded these images by deriving the coding categories inductively from these answers. First, the images were categorized into 36 different categories for cigarettes and 32 for e-cigarettes based on the most frequently occurring images. These categories were further condensed into six categories for cigarettes: synonym, disgust, risky, addiction, satisfaction, and other. The same categories were used for e-cigarettes with addition of “safer/better than cigarettes” and “same/worse than cigarettes” categories. Multiple categories could be assigned to each image; however, use of “other” category was mutually exclusive with the rest of the categories.

The synonym category comprised images dealing with smoke, smoking, physical description of cigarettes and e-cigarettes, brands, and paraphernalia (lighter, ashtray). The disgust category included images and words dealing with repulsed feelings (e.g., “yuck!”), negative perceptions of tobacco users, negative feelings (bad and its synonyms), and derogations (stupid, ridiculous).

The risky category was assigned to images mentioning negative health outcomes of using the product (e.g., lung cancer, death), dangerous chemicals (poison, tar), and concerns about others (bystanders, animals). Addiction images dealt with perceptions of dependence and included mentions of nicotine, need, want, and relief of cravings. Satisfaction included mentions of relief, enjoyment, pleasure, and other positive sensory associations (good taste). For e-cigarettes, two more categories were used. Safer/better comprised images that favorably compared e-cigarettes to cigarettes in terms of health effects, convenience, or dependence. Same/worse included descriptions of e-cigarettes as same as cigarettes or worse in terms of health effects or satisfaction. The category designated "other" included images that did not fit in any of the preceding categories. Examples include mentions of specific people (“My mom”), places (“outside”), times (“1980s”), and complementary consumables (“coffee”) associated with these products, cost, personal stories (e.g., “I quit”, “I don’t smoke”), mentions of taste and smell (when they did not have negative or positive connotation), and “don’t know”, “nothing”, etc.

A reliability check was performed by having a second coder independently categorize a randomly selected set of 10% of images. Intercoder reliability was acceptable [50] (Krippendorf’s alpha, for cigarettes: synonym 0.93, disgust 0.90, risky 0.95, addiction 0.79, satisfaction 0.76, other 0.72; for e-cigarettes: synonym 0.91, disgust 0.68, risky 0.92, addiction 0.90, satisfaction 0.80, safer 0.78, same/worse 0.81, other 0.70).

The most frequent images that non-smokers and former smokers envisioned when hearing the word “cigarette” belonged to the category of risky, followed by disgust (Table 1). In contrast, smokers most frequently mentioned synonyms of smoking, followed by the “other” category (which typically included images of specific people or places associated with cigarettes). These two rather bland and non-specific categories accounted for about 59% of the images offered by current smokers.

**Table 1:** Percentages of participants reporting various images of cigarettes and electronic cigarettes by cigarette smoker status

	Cigarette images				E-cigarette images			
% who mentioned	Total (*n* = 5389)	Current smoker (*n* = 1184)	Former smoker (*n* = 1520)	Never smoker (*n* = 2683)	Total	Current smoker	Former smoker	Never smoker
Synonym	17.5	32.8	16.4	14.2	19.6	24.5	18.7	18.8
Disgust	23.0	6.8	21.7	27.8	13.5	6.0	13.9	15.2
Risky	35.4	13.7	34.6	41.4	11.0	3.5	10.9	12.9
Addiction	4.9	9.5	5.5	3.4	3.9	2.3	4.3	4.0
Relaxing/ Satisfaction	2.6	12.6	2.1	0.3	1.2	2.3	0.8	1.2
Safer than cigarettes	–	–	–	–	5.3	6.2	4.7	5.3
Same/worse than cigarette	–	–	–	–	3.8	2.4	3.2	4.4
Other	18.3	26.1	20.8	15.1	45.6	54.7	47.2	42.5

Images of e-cigarettes were even more extreme in this way. Overall, more than 65% were either synonyms or “other”, the latter being 45.6% (compared to 18.3% for cigarettes. Among current smokers this vacuous imagery was even more pronounced, with 54.7% in the “other” category and 24.5% being synonyms. The most common feelings associated with e-cigarettes were disgust and risky, particularly among former smokers or never smokers, though these two negative images were far less frequent overall (24.5%) then they were for cigarettes (58.4%). For every smoker group, images of e-cigarettes being safer or better than cigarettes were more frequent than images of e-cigarettes being the same or worse than cigarettes.

For the images of cigarettes, offered by current and former e-cigarette users, synonyms were most frequently mentioned, followed by risky (Table 2). For never e-cigarette users, risky and disgust were the most frequent images of cigarettes. For the images of e-cigarettes, regardless of the user status, by far the most frequent images were “other” (45.6%) followed by synonyms (19.6%). Risk as an image was only relatively frequently mentioned by the never users of e-cigarettes (12.7%), while relaxing/satisfaction was mentioned by 6.2% of current e-cigarette users. Regardless of whether participants used e-cigarettes, images of e-cigarettes being safer or better than cigarettes were more frequent than images of e-cigarettes being the same or worse than cigarettes. This was particularly true for current users of e-cigarettes.

**Table 2:** Proportions of participants reporting various images of cigarettes and electronic cigarettes by e-cigarette user status

	Cigarette images				E-cigarette images			
% who mentioned	Total (*n* = 5387)	Current user (*n* = 550)	Former user (*n* = 655)	Never user (*n* = 4182)	Total	Current user	Former user	Never user
Synonym	17.5	32.4	25.8	14.8	19.6	29.0	27.2	17.6
Disgust	23.0	15.9	18.5	24.4	13.5	2.8	9.3	15.2
Risky	35.4	19.8	24.8	38.5	11.0	3.6	4.1	12.7
Addiction	4.9	7.1	6.9	4.4	3.9	1.8	2.8	4.2
Relaxing/ Satisfaction	2.6	8.1	6.2	1.5	1.2	6.2	1.5	0.7
Safer than cigarettes	–	–	–	–	5.3	7.9	5.4	5.0
Same/worse than cigarette	–	–	–	–	3.8	0.6	2.6	4.3
Other	18.3	18.5	18.5	18.3	45.6	48.4	49.2	44.8

Overall, images of cigarettes were valenced more negatively than e-cigarettes, and for both products, current smokers/users had the least negative image, followed by former smokers/users, and never smokers/users had the most negative images (Tables 3 and 4).

**Table 3:** Mean valence of cigarette and electronic cigarette image by cigarette smoker status

	Cigarette images				E-cigarette images			
Image categories	Total	Current smoker	Former smoker	Never smoker	Total	Current smoker	Former smoker	Never smoker
Synonym	− 0.62	0.01	− 0.72	− 1.02	− 0.27	0.19	− 0.19	− 0.47
Disgust	− 0.99	− 0.96	− 0.85	− 1.05	− 0.69	− 0.63	− 0.34	− 0.86
Risky	− 1.40	− 1.42	− 1.32	− 1.43	− 1.22	− 0.66	− 1.11	− 1.30
Addiction	− 0.94	− 0.33	− 1.04	− 1.29	− 0.86	− 0.11	− 0.62	− 1.11
Relaxing/ Satisfaction	1.10	1.30	0.77	0.28	0.96	0.85	1.15	0.94
Safer than cigarettes	–	–	–	–	0.82	1.12	0.72	0.78
Same/worse than cigarette	–	–	–	–	− 0.44	0.25	− 0.34	− 0.57
Other	0.02	− 0.16	0.37	− 0.34	0.03	0.28	0.17	− 0.14
Overall	− 0.83	− 0.05	− 0.70	− 1.10	−0.24	0.22	− 0.10	− 0.43

Note: Valence was rated on a 5-point scale from −2 (very bad) to + 2 (very good) (with 0 being “neither good nor bad”)

**Table 4:** Mean valence of cigarette and electronic cigarette image by electronic cigarette user status

	Cigarette images				E-cigarette images			
Image categories	Total	Current user	Former user	Never user	Total	Current user	Former user	Never user
Synonym	− 0.62	− 0.15	− 0.53	− 0.75	− 0.27	0.72	0.06	− 0.51
Disgust	− 0.99	− 0.62	− 1.04	− 1.01	− 0.69	− 0.37	− 0.93	− 0.65
Risky	− 1.40	− 1.56	− 1.58	− 1.37	− 1.22	− 1.15	− 0.58	− 1.24
Addiction	− 0.94	− 1.02	− 0.72	− 0.97	− 0.86	0.51	− 0.08	− 1.00
Relaxing/Satisfaction	1.10	1.25	1.03	1.07	0.96	1.32	0.97	0.61
Safer than cigarettes	–	–	–	–	0.82	1.30	1.11	0.70
Same/worse than cigarette	–	–	–	–	− 0.44	0.04	0.01	− 0.48
Other	0.02	0.18	0.33	− 0.04	0.03	0.79	0.01	− 0.06
Overall	− 0.83	− 0.38	− 0.64	− 0.91	− 0.24	0.71	0.02	− 0.37

Note: Valence was rated on a 5-point scale from −2 (very bad) to + 2 (very good) (with 0 being “neither good nor bad”)

### Measurement (CFA) models

Results from the CFA models supported the factorial validity of the *affect* and *risk perceptions* scales. The *affect* CFA model was specified with one factor representing affect towards cigarettes and the other factor representing affect towards e-cigarettes. The two affect valence scores for cigarettes and e-cigarettes were loaded separately on the cigarette affect factor and e-cigarette affect factor, respectively. Acceptable model fit was obtained for this model [χ^2^(1) = 5.1, *p* = .02; RMSEA = .028, 95% CI = .008–.055; CFI = 1.0], and all standardized factor loadings exceeded .9. More positive feelings towards cigarettes were associated with more positive feelings toward e-cigarettes as reflected by a positive correlation between the factors (*r* = .63, 95% CI = .60–.66). The *risk perceptions* CFA also fit well by conventional approximate fit standards [χ^2^(19) = 108.0, *p* < .001; RMSEA = .030, 95% CI = .025–.036; CFI = 1.0], with all standardized factor loadings exceeding .95. Higher perception of risks for cigarettes was strongly correlated with perception of risks for e-cigarettes (*r* = .65, 95% CI = .63–.68). Finally, the combined, correlated factors CFA that merged CFA models for *affect* and *risk perceptions* for both products also fit the data well by approximate fit standards [χ^2^(48) = 139.5, *p* < .001; RMSEA = .019, 95% CI = .015–.023; CFI = 1.0]. More positive affect towards cigarettes and towards e-cigarettes was significantly associated with lower perceptions of risk from smoking (*r*s = − .27 and − .19, respectively) and from using e-cigarettes (*r* = − .18 and − .36, respectively).

### Mediation models of affect, risk perceptions, and use

#### Cigarette model

**Fig. 1:**
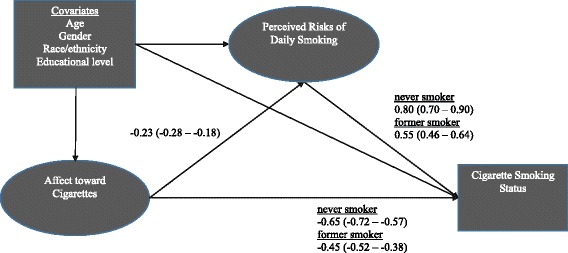
Standardized path coefficient estimates for mediational model of affect towards cigarettes, risk perceptions, smoking status (*n* = 5389). Note: Ovals denote latent factors and rectangles denote observed variables in the model. For visual clarity, only the structural model is shown; the measurement model for the latent factors is not shown. Path coefficient estimates were standardized on the variances of the latent factors only. All estimates are statistically significant (*p* < 0.001). Confidence intervals (95%) are shown in parentheses. Referent group for smoking status was current smokers

Figure 1 depicts the (partially) standardized coefficient estimates for the paths of focal interest to this study (Table 5 provides the unstandardized path coefficient estimates for all paths of the hypothesized structural model). Results were consistent with our hypothesis that affect towards cigarettes predicts smoking status and that this association is partially mediated by risk perceptions: more positive affect towards cigarettes was associated with lower perceived risks of smoking cigarettes, which in turn was associated with higher odds of being a current smoker. Specifically, a 1 *SD* difference in affect towards cigarettes was associated with a 0.23 *SD* difference in perceived risks of smoking after adjustment for the covariates. In turn, a 0.23 SD decrease in perceived risks of smoking was associated with an adjusted 20% higher odds (aOR = *e*^-0.23*-0.80^ = 1.20) of being a current smoker versus being a never smoker (and a 13% greater adjusted odds of being a current smoker vs. a former smoker). Affect towards cigarettes also had a direct effect on smoking status, independent of risk perceptions and covariates: specifically, more positive affect was directly associated with higher odds of being a current smoker (aOR = 1.91 vs. never smoker and aOR = 1.57 vs. former smoker for a 1 SD difference in affect).

**Table 5:** Unstandardized path coefficients for the mediation model of affect towards cigarettes, risk perceptions, and smoking (*n* = 5389)

Direct paths	Path coefficient^a^	95% CI	*p*
Affect → Smoking Status (ref = Current Smoker)			
Never Smoker (vs. Current Smoker)	− 0.20	− 0.22 – − 0.18	< 0.001
Former Smoker (vs. Current Smoker)	− 0.14	− 0.16 – − 0.12	< 0.001
Affect → Risk Perceptions	− 0.66	−0.82 – − 0.50	< 0.001
Risk Perceptions → Smoking Status (ref = Current Smoker)			
Never Smoker (vs. Current Smoker)	0.09	0.08 – 0.09	< 0.001
Former Smoker (vs. Current Smoker)	0.06	0.05 – 0.07	< 0.001
Covariates → Affect			
Age (ref = 18–29 years old)			
30–44 years old	0.49	0.10 – 0.88	0.013
45–59 years old	0.36	− 0.02 – 0.74	0.065
60+ years old	1.16	0.79 – 1.54	< 0.001
Race/Ethnicity (ref = White, non-Hispanic)			
Black, non-Hispanic	0.04	− 0.42 – 0.49	0.872
Hispanic, any race	0.07	− 0.32 – 0.46	0.732
Other	0.28	− 0.30 – 0.87	0.343
Education (ref = <High School)			
High School	− 0.37	− 0.90 – 0.16	0.174
Some College	− 0.72	−1.27 – − 0.16	0.012
Bachelor’s degree or higher	− 1.21	− 1.79 – − 0.64	< 0.001
Female	− 0.50	− 0.78 – − 0.22	< 0.001
Covariates → Risk Perceptions			
Age (ref = 18–29 years old)			
30–44 years old	0.75	− 0.45 – 1.94	0.221
45–59 years old	1.75	0.57 – 2.92	0.004
60+ years old	2.96	1.80 – 4.11	< 0.001
Race/Ethnicity (ref = White, non-Hispanic)			
Black, non-Hispanic	− 0.10	− 1.66 – 1.47	0.902
Hispanic, any race	0.71	− 0.48 – 1.91	0.241
Other	0.02	− 1.86 – 1.91	0.980
Education (ref = <High School)			
High School	1.55	0.03 – 3.07	0.045
Some College	0.88	− 0.67 – 2.43	0.266
Bachelor’s degree or higher	2.42	0.92 – 3.91	0.002
Female	2.11	1.28 – 2.94	< 0.001
Covariates → Never Smoker (vs. Current Smoker)			
Age (ref = 18–29 years old)			
30–44 years old	− 0.68	− 0.88 – − 0.48	< 0.001
45–59 years old	− 0.57	− 0.75 – − 0.38	< 0.001
60+ years old	− 0.02	− 0.23 – 0.20	0.894
Race/Ethnicity (ref = White, non-Hispanic)			
Black, non-Hispanic	− 0.42	− 0.68 – − 0.17	0.001
Hispanic, any race	0.07	− 0.15 – 0.29	0.560
Other	0.27	0.06 – 0.47	0.012
Education (ref = <High School)			
High School	0.31	0.05 – 0.58	0.019
Some College	0.79	0.52 – 1.06	< 0.001
Bachelor’s degree or higher	1.72	1.46 – 1.99	< 0.001
Female	0.02	− 0.12 – 0.17	0.746
Covariates Former smoker (vs. Current Smoker)			
Age (ref = 18–29 years old)			
30–44 years old	0.40	0.15 – 0.65	0.002
45–59 years old	0.75	0.53 – 0.98	< 0.001
60+ years old	1.84	1.58 – 2.09	< 0.001
Race/Ethnicity (ref = White, non-Hispanic)			
Black, non-Hispanic	− 0.67	− 0.93 – − 0.41	< 0.001
Hispanic, any race	− 0.10	− 0.33 – 0.12	0.373
Other	− 0.34	− 0.64 – − 0.04	0.027
Education (ref = <High School)			
High School	0.46	0.16 – 0.76	0.003
Some College	0.72	0.41 – 1.03	< 0.001
Bachelor’s degree or higher	0.88	0.57 – 1.19	< 0.001
Female	− 0.29	− 0.45 – − 0.14	< 0.001
Factor Loadings			
Risk Perceptions			
Lung Cancer	0.98	0.98 – 0.99	< 0.001
Lung Disease (e.g., COPD)	0.99	0.98 – 0.99	< 0.001
Heart Disease	0.97	0.96 – 0.97	< 0.001
Early/Premature Death	0.96	0.95 – 0.97	< 0.001
Affect			
First Image Rating	0.87	0.86 – 0.89	< 0.001
Second Image Rating	1.00	1.00 – 1.00	< 0.001

Ref = referent group or category. **→** denotes an estimated model path (for example, “Affect**→** Smoking Status” signifies the path for the regression of smoking status on affect). ^a^Coeffients are either multinomial logistic coefficients (unstandardized; akin to logistic regression coefficients) if smoking status is the outcome variable (i.e., “**→**Smoking Status”, “**→**Former smoker”, or “**→** Never smoker”), standardized factor loadings or residual correlations for the measurement model paths, or are linear coefficients (akin to regression coefficients) otherwise (i.e., “**→**Affect” or “**→** Risk Perceptions”)

#### E-cigarette model

**Fig. 2:**
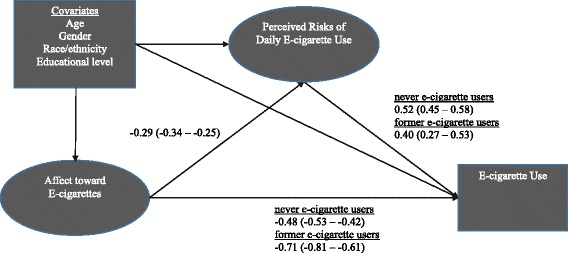
Standardized path coefficients for structural model of affect towards e-cigarettes, risk perceptions, and e-cigarette use (*n* = 5389). Note: Ovals denote latent factors and rectangles denote observed variables in the model. For visual clarity, only the structural model is shown; the measurement model for the latent factors is not shown. Path coefficient estimates were standardized on the variances of the latent factors only. All estimates are statistically significant (*p* < 0.001). Confidence intervals (95%) are shown in parentheses. Referent group for e-cigarette use was current e-cigarette users

Figure 2 depicts the (partially) standardized coefficient estimates for the paths of focal interest to this study (Table 6 provides the unstandardized path coefficient estimates for all paths of the hypothesized structural model). Similar to the results for the cigarette model, results were consistent with our hypothesis that feelings towards e-cigarettes predict e-cigarette use and that this association is partially mediated by risk perceptions towards e-cigarettes: more positive affect towards e-cigarettes was associated with lower perceived risks of using e-cigarettes daily, which in turn was associated with higher odds of being a current e-cigarettes user. Specifically, a 1 *SD* difference in affect towards cigarettes was associated with a 0.29 *SD* difference in perceived risks of e-cigarettes use after adjustment for the covariates. In turn, a 0.29 SD decrease in perceived risks of e-cigarettes use was associated with an adjusted 16% higher odds (aOR = *e*^*-0.*29*-0.52^ = 1.16) of being a current e-cigarettes user versus a never user (and a 12% greater adjusted odds of being a current user vs. a former user). Affect towards e-cigarettes also had a direct effect on e-cigarettes use, independent of risk perceptions and covariates: specifically, more positive affect was directly associated with higher odds of being a current e-cigarette user (aOR = 1.62 vs. never user and aOR = 2.03 vs. former user for a 1 SD difference in affect).

**Table 6:** Unstandardized path coefficients for mediation model of affect towards e-cigarettes, risk perceptions, and e-cigarettes use (*n* = 5389)

Direct paths	Path coefficient^a^	95% CI	*p*
Affect → E-cigarette use status (ref = Current e-cigarette user)			
Never user (vs. Current e-cigarette user)	− 0.24	− 0.27 – − 0.21	< 0.001
Former user (vs. Current e-cigarette user)	− 0.17	− 0.20 – − 0.14	< 0.001
Affect → Risk Perceptions	− 1.05	− 1.28 – − 0.83	< 0.001
Risk Perceptions → E-cigarette use status (ref = Current e-cigarette user)			
Never user (vs. Current e-cigarette user)	0.07	0.06 – 0.08	< 0.001
Former user (vs. Current e-cigarette user)	0.03	0.02 – 0.04	< 0.001
Covariates → Affect			
Age (ref = 18–29 years old)			
30–44 years old	− 0.04	− 0.39 – 0.31	0.818
45–59 years old	− 0.27	− 0.62 – 0.07	0.119
60+ years old	0.14	− 0.19 – 0.48	0.401
Race/Ethnicity (ref = White, non-Hispanic)			
Black, non-Hispanic	0.00	− 0.45 – 0.45	0.994
Hispanic, any race	− 0.39	− 0.75 – − 0.02	0.036
Other	− 0.03	− 0.49 – 0.43	0.899
Education (ref = <High School)			
High School	− 0.18	− 0.62 – 0.25	0.408
Some College	− 0.57	− 1.02 – − 0.13	0.012
Bachelor’s degree or higher	− 0.84	− 1.28 – − 0.40	< 0.001
Female	− 0.26	− 0.50 – − 0.02	0.035
Covariates → Risk Perceptions			
Age (ref = 18–29 years old)			
30–44 years old	1.98	0.71 – 3.25	0.002
45–59 years old	3.39	2.09 – 4.70	< 0.001
60+ years old	5.76	4.36 – 7.16	< 0.001
Race/Ethnicity (ref = White, non-Hispanic)			
Black, non-Hispanic	0.86	− 0.91 – 2.63	0.339
Hispanic, any race	− 0.24	− 1.62 – 1.14	0.736
Other	1.13	− 0.57 – 2.82	0.193
Education (ref = <High School)			
High School	− 0.03	− 1.66 – 1.60	0.969
Some College	0.28	− 1.29 – 1.86	0.723
Bachelor’s degree or higher	0.58	− 0.91 – 2.06	0.446
Female	1.96	1.02 – 2.91	< 0.001
Covariates → Never user (ref = Current e-cigarette user)			
Age (ref = 18–29 years old)			
30–44 years old	0.33	0.07 – 0.58	0.011
45–59 years old	0.90	0.68 – 1.12	< 0.001
60+ years old	1.86	1.61 – 2.11	< 0.001
Race/Ethnicity (ref = White, non-Hispanic)			
Black, non-Hispanic	0.12	− 0.21 – 0.44	0.476
Hispanic, any race	− 0.48	− 0.71 – − 0.24	< 0.001
Other	− 0.29	− 0.66 – 0.08	0.124
Education (ref = <High School)			
High School	0.31	0.04 – 0.57	0.023
Some College	0.48	0.21 – 0.75	< 0.001
Bachelor’s degree or higher	1.15	0.84 – 1.47	< 0.001
Female	− 0.05	− 0.23 – 0.13	0.589
Covariates Former user (ref = Current e-cigarette user)			
Age (ref = 18–29 years old)			
30–44 years old	0.14	− 0.14 – 0.42	0.338
45–59 years old	0.20	− 0.04 – 0.45	0.107
60+ years old	0.45	0.16 – 0.74	0.002
Race/Ethnicity (ref = White, non-Hispanic)			
Black, non-Hispanic	0.26	− 0.07 – 0.59	0.119
Hispanic, any race	− 0.18	− 0.46 – 0.10	0.200
Other	− 0.29	− 0.72 – 0.14	0.182
Education (ref = <High School)			
High School	− 0.01	− 0.30 – 0.28	0.966
Some College	0.31	0.01 – 0.62	0.045
Bachelor’s degree or higher	0.39	0.05 – 0.74	0.027
Female	0.08	− 0.12 – 0.28	0.432
Factor Loadings			
Risk Perceptions			
Lung Cancer	0.99	0.99 – 0.99	< 0.001
Lung Disease (e.g., COPD)	0.99	0.98 – 0.99	< 0.001
Heart Disease	0.97	0.97 – 0.98	< 0.001
Early/Premature Death	0.96	0.96 – 0.97	< 0.001
Affect			
First Image Rating	0.87	0.85 – 0.88	< 0.001
Second Image Rating	1.00	1.00 – 1.00	< 0.001

Ref = referent group or category. **→** denotes an estimated model path (for example, “Affect**→** E-cigarette use status” signifies the path for the regression of e-cigarette use status on affect). ^a^Coeffients are either multinomial logistic coefficients (unstandardized; akin to logistic regression coefficients) if e-cigarette use status is the outcome variable (i.e., “**→**E-cigarette use status”, “**→**Former user”, or “**→** Never user”), standardized factor loadings or residual correlations for the measurement model paths, or are linear coefficients (akin to regression coefficients) otherwise (i.e., “**→**Affect” or “**→** Risk Perceptions”)

## Discussion

In exploring the question of what predicts initiation, maintenance, or cessation of tobacco use, researchers have most frequently provided a rationalistic explanation, focusing on perceptions of risks and benefits of tobacco use, defining them as reasoned assessments or expectancies [8]. More recently, affect or feelings of risk started gaining a more prominent role. In our study, we used a nationally representative sample of U.S. adults to explore how they feel about cigarettes and e-cigarettes, and what attributes of each product are most prominently featured in their affective imagery for these tobacco products. We tested a model in which feelings about a tobacco product were associated with current use of this product by linking with the behavior directly and indirectly through perceptions of risk.

We found that when asked to describe images that first come to their minds when they hear word “cigarettes” participants’ common images reflect disgust and risk. This was somewhat less the case for e-cigarettes whose images were primarily just the descriptions or synonyms of the products, as well as associations with specific people, places, and times, or personal stories related to these products.

Images of cigarettes were strongly negative for former and never smokers, but sometimes positive (e.g. for relaxing), particularly among current smokers. The negatively valenced categories focused on health risks and disgust were particularly frequent for former and never smokers. Images of addiction were rare, and most frequently brought up by current smokers, perhaps because addiction for them is a more salient and visceral experience.

Comparing cigarettes and e-cigarettes, the images for cigarettes were far more negative. Images related to risk and disgust were frequently associated with cigarettes (35% and 23% respectively), but were much less common for e-cigarettes (11% and 14%). While few participants mentioned images in which e-cigarettes were explicitly compared to cigarettes, most of those comparisons were more favorable towards e-cigarettes, with participants mentioning that e-cigarettes were safer, cleaner, or healthier than cigarettes. Smokers who supplied images of e-cigarettes being worse than cigarettes typically held very positive feelings towards cigarettes. For example, one participant wrote about an e-cigarette “Seems too unnatural. I've never really been interested. The thing I like about smoking is that it's earthy and cozy, like a tiny little campfire... there's nothing like that about e-stuff.” Our finding that some participants perceived e-cigarettes as worse than cigarettes because e-cigarettes are “unnatural” resonates with findings from other studies where participants thought that e-cigarettes are more dangerous than “natural” tobacco and marijuana products because e-cigarettes and vaporizers contain various “chemicals” [51].

With regard to e-cigarettes, perhaps the most important finding is the high frequency of the “other” category and synonyms. This suggests that the attitudes of our respondents are rather unformed, cognitively and emotionally, with regard to these new products. This fits with other recent survey data in which questions about risks and other consequences of using e-cigarettes had an unusually high proportion of “don’t know” answers [52].

Campaigns discouraging smoking should reinforce the images focused on risk and disgust, since those were the images that current smokers rated as most negative in valence and they were far less frequent among smokers. This strong negative affect is consistent with findings that most current smokers are miserable and regret having started smoking and want to quit [53, 54]. These findings further support the need to run communication campaigns focused on negative health effects of smoking, such as CDC’s Tips From Former Smokers [55, 56]. Although we did not code for it specifically, quite a few smokers referred to this campaign when they described the first images associated with cigarettes (e.g., “lady on TV with a hole in her throat”). This is in contrast to very few associations with positive images from tobacco advertisements (e.g., “Virginia Slim cigarette ad with the beautiful lady dressed eloquently”). CDC’s Tips campaign has been successful in increasing perceptions of risks and attempts at quitting [56, 57]. However, evaluations of this campaign have not examined to what extent exposure to this campaign has changed smokers’ feelings about smoking and the associated affective imagery, although this might be the mechanism through which it worked. Future studies should explicitly evaluate effects of communication campaigns on affective imagery of tobacco users.

These findings also have implications for the FDA regulations on warning labels. The Congress mandated the FDA to create pictorial warning labels for cigarettes [58]. Pictorial warning labels on cigarettes are required by law in at least 105 countries [59], and extensive research shows that large pictorial warning labels are effective in informing consumers about the harms of smoking and motivating smokers to quit [60–62]. The first set of the pictorial warning labels proposed by the FDA was struck down in court, partially because the court concluded that there was no evidence that the emotional labels selected by the FDA have an effect on reducing smoking [63]. When the FDA proposes its next set of pictorial warning labels, the agency can argue that pictorial warning labels are instrumental in helping achieve the substantial governmental goal of reducing smoking (assuming the case again will be held up to the test of *Central Hudson*) [64], because affect is central to smokers’ decisions to start or quit smoking and that affective messages are needed to break the positive associations smokers have of cigarettes. The association between affect and smoking provides additional evidence to use pictorial warning labels on cigarettes that evoke strong negative emotions.

Over the past several years, comparative perceptions of risks of e-cigarettes in relation to cigarettes have been increasing among adults in the U.S. [52] At the same time, the rates of use of e-cigarettes have been increasing [65, 66]. This is counterintuitive, since perceived risk is usually negatively related to behavior, as described by various health behavior theories [5, 67, 68] and shown empirically for variety of substance use, from marijuana to cigarettes [69, 70]. Our findings provide one possible explanation for this discrepancy – while perceived risk plays a role in e-cigarette use, affect towards e-cigarettes explains current e-cigarette use above and beyond perceptions of risk. We found that on average, adults have more negative affect towards cigarettes than e-cigarettes.

### Limitations

This study employed only two images for each product, in contrast to past studies that used 5–6 images [27, 44, 71]. This might have restricted the pool of affective cues. However, in past studies [44, 71] and this study, the affective valences of the subsequent images were highly correlated, indicating that the first two images might be enough to capture the central affect towards the product.

All measures were self-reported; however, self-report of behavior in surveys has been a reliable measure [72]. Since this was a cross-sectional survey, all data are correlational and, therefore, casual inference is limited. We are limited to concluding only that our data were consistent with our hypothesized and theory-guided mediational model, and acknowledge that alternative models with different causal assumptions (e.g., bi-directional relationships) may also fit the data equally as well. Our study design cannot evaluate which among the current and many alternative models is the correct or best fitting model. Future studies should explore longitudinal effects of affect on behavior or manipulate affect directly as was done by Finucane et al. [26] There could be dynamic feedback loops whereby the experience of tobacco use influences subsequent affect, perceptions of risk, and future tobacco use (e.g., affect→perceptions→product use→affect→…). However, our study was not designed to test dynamic feedback loops. Future intensive longitudinal studies, for example, using ecological momentary assessment (EMA), can evaluate such recursive models. Our study provides initial support to justify these more resource-intensive EMA studies, which would be able to better test and compare the alternative models.

Affect was measured by asking participants to write down an image that comes to mind when they hear the word “cigarettes” or “EVPs”. This open-ended measure provided in-depth insight into visceral feelings participants associate with tobacco products. However, for many participants, the images were not very informative and consisted of the image of the product themselves (i.e., synonyms). To more systematically study affect, future studies should use a more structured approach to soliciting images. Instead of asking participants to write down the first few images of tobacco products, studies should give participants a list of images and ask to what extent they associate these images with each tobacco product and the image’s valence Although the solicitation of images might appear to be visually focused, the instructions did not discourage participants from providing responses related to senses of smell, taste, and touch.

## Conclusion

Our work adds to the limited literature on the association between affect, risk perceptions, and tobacco use by evaluating them for the first time in the nationally representative sample of the U.S. adults. Consistent with the affect heuristic [21], affect towards cigarettes and e-cigarettes was associated with cigarette smoking and e-cigarettes use respectively, and these associations were both direct and partially mediated by risk perceptions towards smoking and e-cigarettes. More positive affect towards tobacco products was associated with lower perceived risks, which in turn was associated with higher odds of being a current tobacco user. The overall relationships between affect, risk perceptions, and current use were similar for cigarettes and e-cigarettes. In developing models explaining tobacco use behavior, or in creating public communication campaigns aimed at curbing tobacco use, it is useful to focus not only on the reason based predictors, such as perceptions of risks and benefits, but also on affective predictors. The former may be very useful in providing a part of the answer, but including affect in the models and looking at the combined effects of reason and affect will enable researchers and regulatory agencies to better understand predictors of use and to design effective communication campaigns.

## Additional files

Additional file 1: **Table S1.** Prevalence of cigarette smoking and e-cigarette use. (DOCX 14 kb)

Additional file 2: **Table S2.** Means of risk rating scores. (DOCX 13 kb)

## Abbreviations

aOR: adjusted odds ratio
CFA: Confirmatory factor analysis
e-cigarettes: electronic cigarettes
EVP: Electronic vapor product
RMSEA: Root mean square error of approximation
SEM: Structural equation modeling
TPRPS: Tobacco Products and Risk Perceptions Survey
WLSMV: mean- and variance-adjusted weighted least squares estimator

## References

[1] United States Department of Health and Human Services (2014). The health consequences of smoking—50 years of progress: a report of the surgeon General.

[2] Jha P, Peto R (2014). Global effects of smoking, of quitting, and of taxing tobacco. N Engl J Med.

[3] Jamal A. Current cigarette smoking among adults—United States, 2005–2015. MMWR Morb Mortal Wkly Rep. 2016;65(44):1205–11. 10.15585/mmwr.mm6544a2 27832052

[4] Slovic P. Smoking: risk, perception, and policy. Sage publications; 2001.

[5] Weinstein ND (1988). The precaution adoption process. Health Psychol.

[6] Bandura A, DiClemente RJ, Peterson JL (1994). Social cognitive theory and exercise of control over HIV infection. Preventing AIDS: Theories and methods of behavioral interventions. Edn.

[7] Slovic P, Finucane M, Peters E, MacGregor DG (2002). Rational actors or rational fools: implications of the affect heuristic for behavioral economics. J Socio-Econ.

[8] Sloan FA, Wang Y (2008). Economic theory and evidence on smoking behavior of adults. Addiction.

[9] Viscusi WK. Smoking: making the risky decision. Oxford university press on demand; 1992.

[10] Henningfield JE, Rose CA, Zeller M (2006). Tobacco industry litigation position on addiction: continued dependence on past views. Tob Control.

[11] Mather L (1998). Theorizing about trial courts: lawyers, policymaking, and tobacco litigation. Law & Social Inquiry.

[12] United States v. Philip Morris, 449 F. Supp. 2d 1 (D.D.C. 2006).

[13] The product in the early 1980s. BAT Co document. Bates No 110069975. [https://www.industrydocumentslibrary.ucsf.edu/tobacco/docs/#id=ggwn0042].

[14] Anderson SJ, Pollay RW, Ling PM (2006). Taking ad-vantage of lax advertising regulation in the USA and Canada: reassuring and distracting health-concerned smokers. Soc Sci Med.

[15] Pollay RW, Dewhirst T (2002). The dark side of marketing seemingly "light" cigarettes: successful images and failed fact. Tob Control.

[16] Epstein S (1994). Integration of the cognitive and the psychodynamic unconscious. Am Psychol.

[17] Slovic P (2012). The ‘value’of smoking: an editorial. Health, risk & society.

[18] Anderson SJ, Glantz SA, Ling PM (2005). Emotions for sale: cigarette advertising and women's psychosocial needs. Tob Control.

[19] Anderson SJ, Dewhirst T, Ling PM (2006). Every document and picture tells a story: using internal corporate document reviews, semiotics, and content analysis to assess tobacco advertising. Tob Control.

[20] Loewenstein GF, Weber EU, Hsee CK, Welch N (2001). Risk as feelings. Psychol Bull.

[21] Slovic P, Finucane ML, Peters E, MacGregor DG (2007). The affect heuristic. Eur J Oper Res.

[22] Damasio AR. Descartes' error: emotion, reason, and the human brain. New York: Grosset/Putnam; 1994.

[23] Trafimow D, Sheeran P, Lombardo B, Finlay KA, Brown J, Armitage CJ (2004). Affective and cognitive control of persons and behaviours. Br J Soc Psychol.

[24] Lawton R, Conner M, McEachan R (2009). Desire or reason: predicting health behaviors from affective and cognitive attitudes. Health Psychol.

[25] Zajonc RB (1980). Feeling and thinking: preferences need no inferences. Am Psychol.

[26] Finucane ML, Alhakami A, Slovic P, Johnson SM. The affect heuristic in judgments of risks and benefits. J Behav Decis Mak. 2000;13(1):1–17.

[27] Marks AD, O'Neill G, Hine DW. Role of affect, expectancies and dual processes of cognition in predicting adult cigarette smoking. Aust J Psychol. 2008;60(3):160–7.

[28] Schutte NS, Marks AD (2013). Smoking status and intention to quit: the role of affective associations and expectancies. J Drug Educ.

[29] Abrams DB (2014). Promise and peril of e-cigarettes: can disruptive technology make cigarettes obsolete?. JAMA.

[30] Fagerstrom K, Etter J-F, Unger JB (2015). E-cigarettes: a disruptive technology that revolutionizes our field?. Nicotine Tob Res.

[31] Stimson GV, Thom B, Uhl A (2014). Disruptive innovations: the rise of the e-cigarette. International Journal of Drug Policy.

[32] Pechacek TF, Nayak P, Gregory KR, Weaver SR, Eriksen MP. The potential that electronic nicotine delivery systems can be a disruptive technology: results from a national survey. Nicotine Tob Res. 2016;18(10):1989–97. 10.1093/ntr/ntw102 PMC5016845 27142201

[33] Fairchild AL, Bayer R, Colgrove J (2014). The renormalization of smoking? E-cigarettes and the tobacco “endgame”. N Engl J Med.

[34] Kalkhoran S, Glantz SA (2016). E-cigarettes and smoking cessation in real-world and clinical settings: a systematic review and meta-analysis. Lancet Respir Med.

[35] Dutra LM, Grana R, Glantz SA. Philip Morris research on precursors to the modern e-cigarette since 1990. Tob Control; 2017;26:e97–e105. 10.1136/tobaccocontrol-2016-053406 PMC5432409 27852893

[36] Mickle T. Reynolds deal could help British American Tobacco make up lost ground: U.S. company would bring with it technology to catch up in race over alternatives to cigarettes. Wall Street J. 2016; http://www.wsj.com/articles/reynolds-deal-could-help-british-american-make-up-lost-ground-1477236062. Accessed 19 Mar 2018.

[37] Grana R, Ling PM (2014). “Smoking revolution”: a content analysis of electronic cigarette retail websites. Am J Prev Med.

[38] Trinkets and Trash: Artifacts of the tobacco epidemic [http://www.trinketsandtrash.org/].

[39] Cooper M, Case KR, Loukas A, Creamer MR, Perry CL (2016). E-cigarette dual users, exclusive users and perceptions of tobacco products. Am J Health Behav.

[40] Wackowski OA, Delnevo CD. Young Adults’ Risk Perceptions of Various Tobacco Products Relative to Cigarettes Results From the National Young Adult Health Survey. Health Educ Behav. 2015;43(3):328–36. 10.1177/1090198115599988 PMC4766060 26304709

[41] Piñeiro B, Correa JB, Simmons VN, Harrell PT, Menzie NS, Unrod M, Meltzer LR, Brandon TH (2016). Gender differences in use and expectancies of e-cigarettes: online survey results. Addict Behav.

[42] Popova L, So J, Sangalang A, Neilands TB, Ling P. Do emotions spark interest in alternative tobacco products? Health Educ Behav. 2017;44(2):598-612. 10.1177/1090198116683169 PMC5494011 28071144

[43] Peters E, Slovic P. The role of affect and worldviews as orienting dispositions in the perception and acceptance of nuclear power. J Appl Soc Psychol. 1996;26(16):1427–53.

[44] Slovic P, MacGregor DG, Peters E. Imagery, affect, and decision making. 1998. https://scholarsbank.uoregon.edu/xmlui/handle/1794/20644.

[45] Muthén LK, Muthén BO (2010). Mplus User's Guide: Statistical Analysis with Latent Variables: User'ss Guide: Muthén & Muthén.

[46] Flora DB, Curran PJ (2004). An empirical evaluation of alternative methods of estimation for confirmatory factor analysis with ordinal data. Psychol Methods.

[47] Hu LT, Bentler PM (1999). Cutoff criteria for fit indexes in covariance structure analysis: conventional criteria versus new alternatives. Struct Equ Model Multidiscip J.

[48] Enders CK. Applied missing data analysis. Guilford press; 2010.

[49] Weaver SR, Kemp CB, Heath JW, Pechacek TF, Eriksen MP. Use of nicotine in electronic nicotine and non-nicotine delivery systems by U.S. adults, 2015. Public Health Rep. 2017;132(5):545–8. 10.1177/0033354917723597 PMC5593242 28880788

[50] Krippendorff K. Content analysis: an introduction to its methodology. Sage; 2004.

[51] Byron MJ, Baig SA, Moracco KE, Brewer NT. Adolescents’ and adults’ perceptions of ‘natural’,‘organic’and ‘additive-free’cigarettes, and the required disclaimers. Tob Control. 2016;25:517–20. 10.1136/tobaccocontrol-2015-052560 PMC4887411 26628496

[52] Majeed BA, Weaver SR, Gregory KR, Whitney CF, Slovic P, Pechacek TF, Eriksen MP. Changing perceptions of harm of E-cigarettes among U.S. adults, 2012-2015. Am J Prev Med. 2017;52(3):331–8. 10.1016/j.amepre.2016.08.039 PMC5373478 28341303

[53] Fong GT, Hammond D, Laux FL, Zanna MP, Cummings KM, Borland R, Ross H. The near-universal experience of regret among smokers in four countries: findings from the International Tobacco Control Policy Evaluation Survey. Nicotine Tob Res 2004, 6(Suppl 3):S341-S351. 10.1080/14622200412331320743 15799597

[54] O'Connor RJ, Thrasher JF, Bansal-Travers M. Exploring relationships among experience of regret, delay discounting, and worries about future effects of smoking among current smokers. Substance use & misuse. 2016;51(9):1245–50. 10.3109/10826084.2016.1160123 PMC5008256 27192133

[55] Tips from Former Smokers [http://www.cdc.gov/tobacco/campaign/tips/].

[56] McAfee T, Davis KC, Alexander RL, Pechacek TF, Bunnell R (2013). Effect of the first federally funded US antismoking national media campaign. Lancet.

[57] Huang L-L, Thrasher JF, Abad EN, Cummings KM, Bansal-Travers M, Brown A, Nagelhout GE. The US National tips from former smokers antismoking campaign promoting awareness of smoking-related risks, cessation resources, and cessation behaviors. Health Educ Behav. 2015;42(4):480–6. 10.1177/1090198114564503 PMC4501900 25588934

[58] Family Smoking Prevention and Tobacco Control and Federal Retirement Reform. Public Law 111–31, U.S. Statutes at Large 123 (2009):1776; http://www.gpo.gov/fdsys/pkg/PLAW-111publ31/pdf/PLAW-111publ31.pdf*.*

[59] Canadian Cancer Society (2016). Cigarette package health warnings: international status report.

[60] Hammond D (2011). Health warning messages on tobacco products: a review. Tob Control.

[61] Noar SM, Hall MG, Francis DB, Ribisl KM, Pepper JK, Brewer NT. Pictorial cigarette pack warnings: a meta-analysis of experimental studies. Tob Control. 2016;25:341–54. 10.1136/tobaccocontrol-2014-051978 PMC4636492 25948713

[62] Noar SM, Francis DB, Bridges C, Sontag J, Ribisl KM, Brewer NT. The impact of strengthening cigarette pack warnings: Systematic review of longitudinal observational studies. Soc Sci Med. 2016;164:118–29. 10.1016/j.socscimed.2016.06.011 PMC5026824 27423739

[63] R.J. Reynolds Tobacco Co. v. FDA, 696 F.3d 1205 (D.C. Cir. 2012).

[64] Central Hudson Gas & Electric Corp. v. Public Service Commission, 447 US 557 (1980).

[65] King BA, Patel R, Nguyen KH, Dube SR (2015). Trends in awareness and use of electronic cigarettes among US adults, 2010–2013. Nicotine Tob Res.

[66] Weaver SR, Majeed BA, Pechacek TF, Nyman AL, Gregory KR, Eriksen MP (2016). Use of electronic nicotine delivery systems and other tobacco products among USA adults, 2014: results from a national survey. International journal of public health.

[67] Rosenstock I (1974). Health belief model and preventive health behavior. Health Educ Monogr.

[68] Ajzen I, Kuhl J, Beckman J (1985). From intentions to actions: a theory of planned behavior. Control: From cognition to behaviors.

[69] Johnston LD, O'Malley PM, Miech RA, Bachman JG, Schulenberg JE (2015). Monitoring the future national survey results on drug use: 1975–2014: overview, key findings on adolescent drug use.

[70] Bachman JG, Johnson LD, O'Malley PM (1998). Explaining recent increases in students' marijuana use: impacts of perceived risks and disapproval, 1976 through 1996. Am J Public Health.

[71] Slovic P, Layman M, Kraus N, Flynn J, Chalmers J, Gesell G (1991). Perceived risk, stigma, and potential economic impacts of a high-level nuclear waste repository in Nevada. Risk Anal.

[72] Caraballo RS, Giovino GA, Pechacek TF (2004). Self-reported cigarette smoking vs. serum cotinine among US adolescents. Nicotine Tob Res.

