# Supplementary material for: Affect, risk perception, and the use of cigarettes and e-cigarettes: a population study of U.S. adults
Source: BMC Public Health. 2018 Mar 22;18:395. doi: 10.1186/s12889-018-5306-z (PMC5863900; doi:10.1186/s12889-018-5306-z)
Supplement: Supplementary file 1 — Table S1. Prevalence of cigarette smoking and e-cigarette use. (DOCX 14 kb) [file 12889_2018_5306_MOESM1_ESM.docx]

**Additional file 1**

**Table S1: Prevalence of cigarette smoking and e-cigarette use**

| 1. **Cigarette smoking status by e-cigarette use status** | | | | |
| --- | --- | --- | --- | --- |
| **Cigarette smoking status** | **Frequency** | **E-cigarette use status** | | |
|  |  | **Current user (%)** | **Former user (%)** | **Never user (%)** |
| Current smoker | 1184 | 32.7 | 26.9 | 40.4 |
| Former smoker | 1520 | 6.0 | 14.6 | 79.4 |
| Never smoker | 2497 | 3.4 | 4.9 | 91.7 |
| **Total** | **5387** | **8.5** | **11.0** | **80.6** |
| 1. **E-cigarette use status by cigarette smoking status** | | | | |
| **E-cigarette use status** | **Frequency** | **Cigarette smoking status** | | |
|  |  | **Current smoker (%)** | **Former smoker (%)** | **Never smoker (%)** |
| Current user | 550 | 56.9 | 20.3 | 22.8 |
| Former user | 655 | 36.3 | 38.2 | 25.5 |
| Never user | 4182 | 7.4 | 28.2 | 64.4 |
| **Total** | **5387** | **14.8** | **28.7** | **56.6** |

Note: The total n does not add up to 5,389 because two participants had missing data.
