# Supplementary material for: Affect, risk perception, and the use of cigarettes and e-cigarettes: a population study of U.S. adults
Source: BMC Public Health. 2018 Mar 22;18:395. doi: 10.1186/s12889-018-5306-z (PMC5863900; doi:10.1186/s12889-018-5306-z)
Supplement: Supplementary file 2 — Table S2. Means of risk rating scores. (DOCX 13 kb) [file 12889_2018_5306_MOESM2_ESM.docx]

Additional file 2

**Table S2: Means of risk rating scores**

| **Type of risk** | **Cigarettes**  **(Mean)** | **E-cigarettes**  **(Mean)** |
| --- | --- | --- |
| Lung cancer | 5.37 | 4.22 |
| Lung disease other than lung cancer | 5.41 | 4.30 |
| Heart disease | 5.30 | 4.18 |
| Early/Premature death | 5.32 | 4.17 |

Note: Scores were on a 0 (“No chance”) to 6 (“Very good chance”) scale.
